# *In vivo* assessment of behavioral recovery and circulatory exchange in the peritoneal parabiosis model

**DOI:** 10.1038/srep29015

**Published:** 2016-07-01

**Authors:** Joseph M. Castellano, Mikael Palner, Shi-Bin Li, G. Mark Freeman, Andy Nguyen, Bin Shen, Trisha Stan, Kira I. Mosher, Frederick T. Chin, Luis de Lecea, Jian Luo, Tony Wyss-Coray

**Affiliations:** 1 Department of Neurology and Neurological Sciences, Stanford University School of Medicine, Stanford, 94305 CA, USA; 2 Department of Radiology, Stanford University School of Medicine, Stanford, 94305 CA, USA; 3 Department of Psychiatry and Behavioral Sciences, Stanford University School of Medicine, Stanford, 94305 CA, USA; 4 Center for Tissue Regeneration, Repair and Restoration, VA Palo Alto Healthcare System, Palo Alto, 94304 CA, USA

## Abstract

The sharing of circulation between two animals using a surgical procedure known as parabiosis has created a wealth of information towards our understanding of physiology, most recently in the neuroscience arena. The systemic milieu is a complex reservoir of tissues, immune cells, and circulating molecules that is surprisingly not well understood in terms of its communication across organ systems. While the model has been used to probe complex physiological questions for many years, critical parameters of recovery and exchange kinetics remain incompletely characterized, limiting the ability to design experiments and interpret results for complex questions. Here we provide evidence that mice joined by parabiosis gradually recover much physiology relevant to the study of brain function. Specifically, we describe the timecourse for a variety of recovery parameters, including those for general health and metabolism, motor coordination, activity, and sleep behavior. Finally, we describe the kinetics of chimerism for several lymphocyte populations as well as the uptake of small molecules into the brains of mice following parabiosis. Our characterization provides an important resource to those attempting to understand the complex interplay between the immune system and the brain as well as other organ systems.

Many advances in immunology, endocrinology, neuroscience, and regenerative medicine have taken advantage of a surgical technique used for well over a century known as parabiosis^1^. In this technique, animals are connected surgically, often along the adjacent flanks, to create shared circulation arising from the newly formed vascular anastomosis. Parabiosis has been used to delineate the role of several humoral factors critical for growth and reproduction^2^, resulting in greater understanding of endocrine signals that act on multiple sites throughout the body to facilitate complex organismal behavior. Coleman and colleagues parabiosed diabetes (*db/db*) mutant mice to wild-type mice in work that ultimately led to identification of leptin as a humoral satiety factor acting on the hypothalamus to regulate energy homeostasis^3^. Though parabiosis provided several critical insights to our understanding of communication between blood and distant organs^4^,^5^, the model has undergone resurgence, especially in regenerative medicine^6^ and neurodegeneration studies^7^,^8^. Reminiscent of mid-20^th^-century investigations that searched for systemic factors regulating lifespan^9^,^10^,^11^,^12^, this line of inquiry was rekindled in 2005 to identify regenerative pathways reactivated by exposure to systemic factors^13^. In this seminal study from the Rando laboratory, Conboy *et al*. compared pairs of the same age to heterochronic pairings of young and old mice, finding that factors in young blood rejuvenate aged skeletal muscle stem cells and regeneration following injury. Aside from muscle^13^,^14^,^15^, several groups have shown that young blood can be restorative for liver^13^, pancreas^16^, heart^17^, bone^18^, brain^19^,^20^,^21^ and spinal cord^22^. For example, heterochronic parabiosis revealed fresh insights that adult neurogenesis and synaptic plasticity in the aged brain may be revitalized by youthful blood-borne cues^6^, arguing for a complex compartmental physiology whose description could only be made using parabiosis as the conceptual nidus. Growth and differentiation factor 11 (GDF11), an anti-aging factor identified using parabiosis, appears to act on heart^17^, muscle^14^, and adult neurogenesis^19^. Though GDF11 activity in muscle and heart and the age-related decrease in blood have been controversial (see Hinken *et al*.^23^, Walker *et al*.^24^, Conboy *et al*.^25^ for recent work or reviews), future studies will clarify the role of GDF11 in aged tissues, its reliable detection, and the conditions under which it exerts its effects. Heterochronic parabiosis was also instrumental in identifying pro-aging factors that stifle adult neurogenesis, including CCL11^20^ and β2-microglobulin^26^ (β2M). A separate study decreased β2M levels in cardiotoxin-injured mice by targeting TGF-β1 signaling, resulting in increased hippocampal neurogenesis as well as elevated skeletal myogenesis^27^. Beyond aging research, the parabiosis model has been used to answer questions ranging from metabolism^28^,^29^,^30^ to ischemic preconditioning^31^ while continuing to be a mainstay in immunology research^32^,^33^. By tracking cellular cross-circulation in parabionts, several groups have addressed controversy over the origin of CNS macrophages^34^, as well as macrophage infiltration in the context of amyloid pathology^8^, peripheral nerve injury^35^, facial nerve injury^36^, or experimental autoimmune encephalitis^37^.

The modern procedure is inspired by earlier versions of the surgical procedure but with adaptations that increase survival and recovery, including enhanced post-operative monitoring, the use of animals of the same genetic background, and suturing the joints together to promote coordinated movement. Despite these improvements, many questions still remain regarding recovery of the animals following parabiosis, hampering acceptance and implementation of the model as well as the design and interpretation of experiments. We sought to characterize how parabionts respond to the surgery in terms of various physiological and behavioral parameters and in terms of how quickly factors in the blood are exchanged. We provide the first description of recovery parameters for the parabiosis model, which will serve as a resource to design experiments that assess complex interactions between the systemic and CNS compartments or other organ systems.

## Materials and Methods

### Animals

Young, male, wild-type C57BL/6J and UBC-GFP C57BL/6J mice were purchased from Jackson Labs and kept on a 12-h light/dark cycle and provided *ad libitum* access to food and water. All animal care and procedures complied with the Animal Welfare Act and were carried out in accordance with institutional guidelines and approved by the VA Palo Alto Committee on Animal Research or the institutional administrative panel of laboratory animal care at Stanford University.

### Parabiosis surgery and recovery monitoring

Parabiosis was performed as described^21^ using the peritoneal method in which small mirror-image incisions are made along adjacent flanks of age-matched mice in order to continuously suture together the peritoneal cavity. Surgeries were performed under aseptic conditions with controlled isoflurane anesthesia. Knee and elbow joints from adjacent mice were joined together with nylon monofilament sutures to promote coordinated locomotion. Surgical autoclips (9-mm, Clay Adams) were used to join the skin together and to limit infection. Surgeries were carried out on heating pads and body temperature was monitored throughout the procedure. Mice in each pair were injected subcutaneously with Baytril antibiotic (5 μg/g) and Buprenorphine as indicated to limit infection and manage pain, supplementing 0.9% (w/v) sodium chloride for hydration. Sham surgeries were identical to parabiosis surgeries except for joining to the other mouse; sham mice were co-housed before and after surgery. Pairs and shams were monitored continuously according to recovery parameters described in previous work^21^,^38^. For corticosterone measurements, serial submandibular bleeds were performed under light and rapid isoflurane anesthesia and EDTA-plasma was collected and used for corticosterone ELISA (Enzo Life Sciences) as described^21^. For serum chemistry and metabolic analysis, blood was collected from mice sacrificed at indicated timepoints, incubating at room temperature for 30 min and centrifuged at 2000 × g for 10 min at 4 °C. Serum samples were analyzed by the Animal Diagnostic Laboratory at the Stanford Veterinary Service Center; metabolites for which hemolysis significantly confounds accurate quantification were not analyzed.

### Activity and rotarod behavioral assays

Velocity and activity measurements were obtained using the SmartCage^TM^ “beam-break” monitoring system in which parabionts or sham mice explored a specialized cage resembling their home cage. Activity counts and velocity in the center (outside of darkened retreat box) was averaged over the initial ten minutes of exploration during the hour-long exposure. Following training trials in which mice habituated to the rotarod task under zero-acceleration conditions over 30 seconds, parabionts or shams were placed on the rotating and accelerating rod for test trials. Shams were run side-by-side to mimic running conditions experienced by parabionts. Running latency was recorded as the time to fall for parabionts or, in the case of shams, when at least one sham in a running sham pair fell. Presurgery baselines were obtained by running co-housed mice side-by-side on rotarod assay.

### Sleep studies

Cortical electroencephalogram (EEG) recordings were sampled through mini-screws placed in the skull above the frontal (AP, −2 mm; ML, ±1 mm) and temporal (AP, 3; ML, ±2.5 mm) cortices. Electromyography (EMG) was recorded from mini-rings connected with insulated wires inserted into neck muscle. Mini-screws/rings and connected mini-electrode sockets were fixed with meta-bond and dental acrylic, after which mice were allowed to recover for at least one week. Following recovery, mice were connected to flexible recording leads, habituated, and EEG/EMG was recorded and analyzed over the 12-hour light phase over which mice are predominantly inactive (sleep). Mice were disconnected and pair-housed for an additional week prior to parabiosis surgery. Recording cables were re-connected to parabionts and EEG/EMG recordings were made at indicated times. EEG/EMG signals were sampled with the VitalRecorder (Kissei Comtec Co.) system equipped with a multiple channel amplifier (Grass Instruments). Filtering for EEG and EMG signals was set between 0.1 and 200 Hz with a sampling frequency of 500 Hz. Raw data recorded by VitalRecorder were analyzed in a MATLAB-based application using custom scripts as well as built-in MATLAB tools (The MathWorks, Inc.). Signals were staged offline based on fast Fourier transform (FFT) and staging criteria for EEG signals as described^39^. A Power spectral density (PSD) function was used to plot mean power-frequency distributions (1–30 Hz), as described by the work of Scheffzük and colleagues^40^. Recordings for some later timepoints were not taken if partner mouse damaged wires significantly.

### Flow cytometry

Young, male WT and GFP mice were joined by parabiosis and allowed to recover, sampling blood by low-volume, submandibular bleeds at indicated postsurgical times for flow cytometry analysis, as described^41^. Briefly, after red blood cell lysis, cells were fixed and permeabilized before staining for cell surface markers with BD Biosciences antibodies: B220 (RA3-6B2), CD4 (GK1.5), and CD8 (53–6.7). An LSR II was used to collect multiparameter flow cytometry data for analysis with FlowJo software (TreeStar).

### FDG-PET

Under isoflurane/oxygen inhalation, a tail vein catheter was inserted in the tail of “mouse A” for each pair, after which a 100-μL saline flush was given to maintain patency before sealing the catheter. Two catheterized and overnight-fasted (to limit competition of [^18^F]-FDG with endogenous glucose for GLUT1) pairs were placed in a customized platform within an Inveon microPET/CT (Siemens) scanner, and body temperature was maintained with an infrared lamp. Each pair received an intravenous injection of [^18^F]-FDG (210 ± 26 μCi) and simultaneous 60-min dynamic PET. Data were histogrammed into 30 frames, each reconstructed using two-dimensional ordered-subset expectation maximization (2DOSEM) with arc and scatter correction; one frame from the final 15-min acquisition was reconstructed using 3DOSEM. No attenuation correction was made due to epidermal autoclips present along the peritoneum. Images were imported into Inveon Research Workspace 4.0 (Siemens), and mean tracer activity over time was measured in coronal ROIs. 3D PET images were used to generate maximum intensity projections (MIP) used as representative images.

### Statistics

Two-group comparisons were made using two-tailed, unpaired Student’s t test. For comparisons of more than two groups, 1-way ANOVA followed by Tukey’s post-hoc test was used. 2-way ANOVA with Tukey’s post-hoc test was used for time X surgery group comparisons in which timepoints were independent. Repeated-measures ANOVA with Sidak’s post-hoc test were used for time X surgery group comparisons with repeated observations. Data were analyzed using GraphPad Prism 6.0 or Stata 13.0 for unbalanced datasets. α = 0.05, and data are expressed as mean ± s.e.m. *P < 0.05, **P < 0.01, ***P < 0.001; ****P < 0.0001.

## Results

### Recovery of general health parameters in parabionts

To characterize the general health of mice undergoing parabiosis, we first examined a panel of basic serum chemistry and metabolic parameters in mice sacrificed at various times following parabiosis surgery (Table 1). With the exception of phosphorus and carbon dioxide, specific electrolytes did not change significantly at any of the timepoints following parabiosis surgery compared to mice with no surgery (hereafter referred to as “baseline”). Serum carbon dioxide levels were higher at the four-week postsurgical timepoint, though similar to levels measured in sera from mice given sham surgeries without parabiosis, indicating mild surgery-associated hypercapnia. Overall liver and kidney function, as assessed by serum levels of albumin, globulin, creatinine and blood urea nitrogen, appeared to be normal at all timepoints studied. Significant changes in total serum protein levels in parabionts quickly recovered to sham or baseline levels.

### Recovery of behavioral parameters weeks after sharing systemic environment

**Figure 1:**
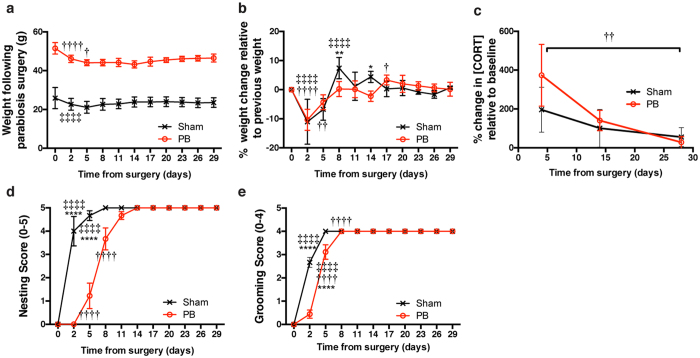
Physiological and behavioral recovery of parabionts. (**A)** Absolute weights or **(B)** percentage change vs. previous weight of pairs (parabionts) or mice given sham surgery measured at various times during recovery (N = 9 pairs; N = 6 sham). **(C)** Percentage change in plasma corticosterone levels vs. baseline (N = 6/group). **(D)** Nesting and **(E)** grooming scores for recovering mice as described previously^21^; (N = 9 pairs; N = 6 sham). *Symbols indicate differences between groups; † or ‡ symbols indicate differences relative to previous timepoint within parabiosis or sham groups, respectively, assessed by repeated-measures ANOVA with Sidak’s post-hoc test for time X surgical group comparisons. One, two, three, or four symbols indicate *P* < 0.05, *P* < 0.01, *P* < 0.001, or *P* < 0.0001, respectively. Values are mean ± SEM.

**Figure 2:**
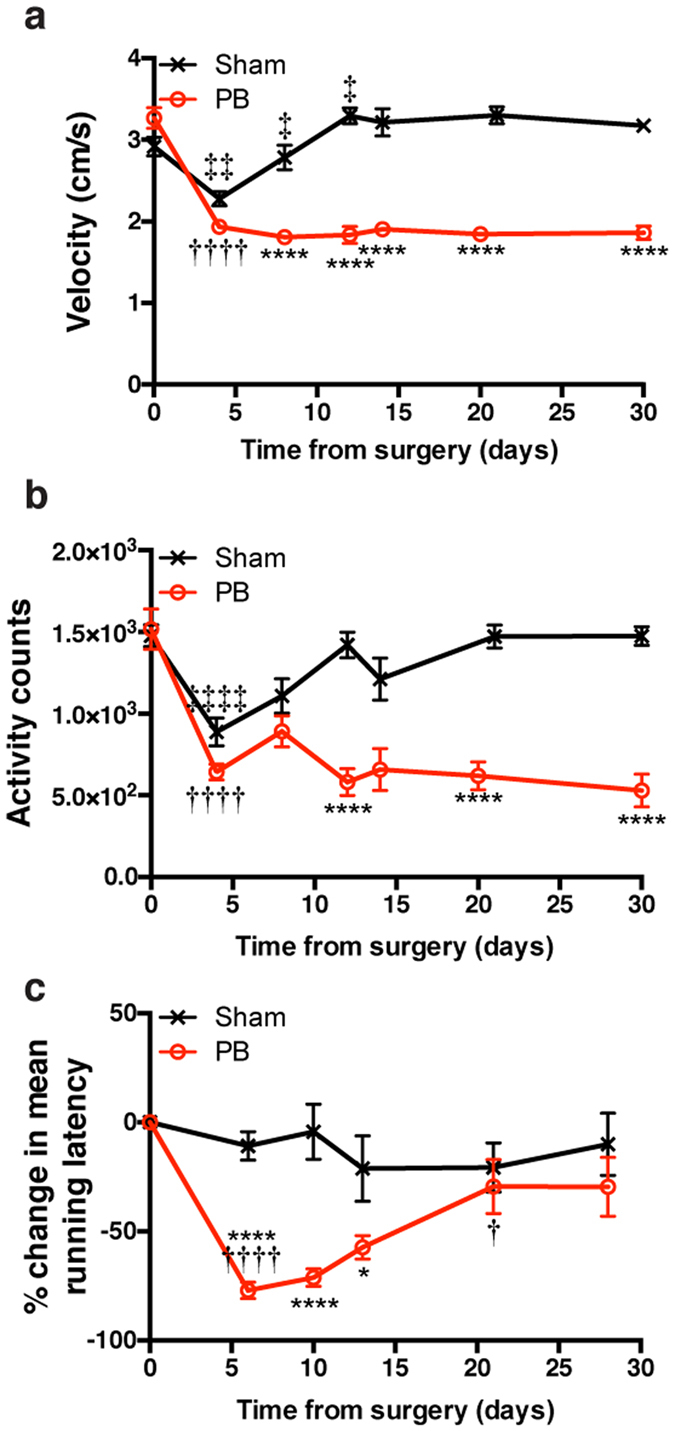
Motor activity and coordination recovery of parabionts. (**A**) Mean velocity and **(B)** activity counts measured during first ten minutes of SmartCage monitoring (N = 9 pairs; N = 8 sham). **(C)** Percentage change from baseline in mean running latency before rotarod slip (N = 9 pairs; N = 4 sham). *Symbols indicate differences between groups; † or ‡ symbols indicate differences relative to previous timepoint within parabiosis or sham groups, respectively, assessed by repeated-measures ANOVA with Sidak’s post-hoc test for time X surgical group comparisons. One, two, three, or four symbols indicate *P* < 0.05, *P* < 0.01, *P* < 0.001, or *P* < 0.0001, respectively. Values are mean ± SEM.

We next sought to assess indicators of physiological and behavioral stress longitudinally in parabionts or mice given sham surgery. Following surgery, both groups appeared to lose weight immediately, which was followed by steady normalization of weight (Fig. 1a). Furthermore, the percentage change in weight over time appeared to be similar between sham and parabiont groups (Fig. 1b). High levels of circulating corticosterone for prolonged periods of time can negatively alter endocrine, immune, and other physiological functions^42^,^43^. To examine the postoperative stress response, we serially sampled plasma for corticosterone measurements from parabionts or mice given sham surgeries. Four days after surgery, plasma corticosterone levels were elevated in both parabionts and sham mice (Fig. 1c), indicating biochemical stress associated with the procedure that ultimately normalized four weeks after the surgery. We next examined stress and general health on a behavioral level by assessing scored nesting and grooming ability^21^,^38^. Both groups exhibited a gradual but complete recovery in nesting and grooming behavior over time, albeit at different rates (Fig. 1d,e). To further investigate behavioral function in parabionts and mice given sham surgeries, we measured overall motor activity and velocity by tracking beam-breaks using the SmartCage^TM^ monitoring system. SmartCage analysis revealed reductions in both velocity and overall activity for both groups (Fig. 2a,b). However, while parabionts came to a lower, but stable velocity and activity level following surgery, sham mice returned to pre-surgery baseline levels. Notably, parabionts trained to run on a rotating, accelerating rod over several trials in the rotarod task exhibited impaired motor coordination that was eventually regained (Fig. 2c); motor coordination in parabionts 21 days after surgery was indistinguishable from that measured in sham animals.

**Figure 3:**
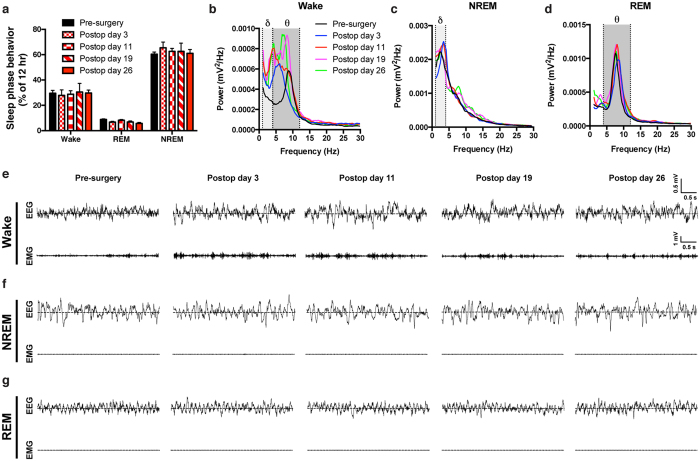
Sleep recovery of parabionts by EEG/EMG recording. (**A)** Wake, REM, and NREM sleep phase behavior measured by EEG/EMG recording in parabionts during pre-surgical and postoperative assessments (N = 3–6 pairs/timepoint). (**B**) Power spectral density (PSD) curves of EEG data during wake, **(C)** NREM, and **(D)** REM periods of the sleep phase in parabionts for the indicated postoperative timepoints compared to the same mice prior to parabiosis surgery. Delta (1–4 Hz) and theta (4–12 Hz) bands are indicated by shading. **(E)** Representative EEG and EMG traces during wake, **(F)** NREM, and **(G)** REM epochs (scale bars indicated), demonstrating appropriate sleep staging. Differences across timepoints were assessed by a mixed repeated-measures model for EEG/EMG data using Stata 13.0 software. Values are mean ± SEM.

Because normal sleep behavior is important for many physiological functions^44^, including those recently under investigation using the parabiosis model^21^, we next assessed sleep behavior before and following parabiosis surgery. Sleep behavior was examined in parabionts implanted with EEG/EMG electrodes during the light phase when mice are predominantly inactive and asleep. Compared to a pre-surgery baseline assessment, the proportion of time spent in wake, REM, and NREM behavioral states was unaffected by the parabiosis procedure (Fig. 3a). To examine sleep behavior in greater detail, we performed a power spectral density analysis on EEG signals using fast Fourier transform during wake, REM, and NREM states of the light phase (Fig. 3b–g). Parabiosis did not affect overall sleep power in both NREM and REM states, and the characteristic delta (1–4 Hz) and theta (4–12 Hz) power bands in NREM (Fig. 3c) and REM (Fig. 3d), respectively, were unaffected, suggesting intact sleep physiology. Interestingly, during the waking state, a period during which voluntary movement-associated theta rhythms are predominant^40^,^45^, we observed an early theta shift towards the delta band. As the parabionts recovered, both theta and delta-like (1–6 Hz) power bands were observed, demonstrating an alternation between voluntary (theta) and involuntary (delta-like) movements as the mice adapt to the physical constraints of parabiosis.

### Kinetics of lymphocyte and small molecule exchange following parabiosis

**Figure 4:**
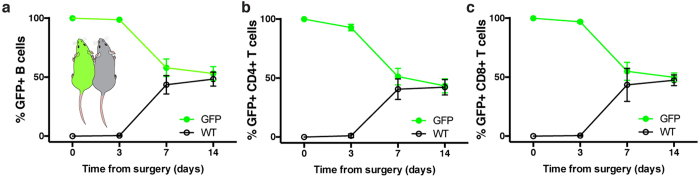
Timecourse of lymphocyte exchange in blood following parabiosis. **(A)** Percentage of GFP+ B cells **(B)** CD4+ T cells, and **(C)** CD8+ T cells measured from the blood of WT or UBC-GFP parabionts at indicated timepoints until chimerism is reached (N = 9 pairs; mean ± SD). Green traces represent serially isolated plasma samples from the GFP mouse in each pair; black traces represent plasma taken from WT mouse in each pair.

We next sought to assess the kinetics of blood exchange of cellular and molecular species at various times following the joining of circulation by parabiosis. To track the migration and equilibration of lymphocytes from one parabiont into its partner’s circulation, we connected age-matched UBC-GFP-transgenic mice to wild-type (WT) mice of the same genetic background (C57Bl/6J). Using flow cytometry to count the number of GFP+ leukocytes of the B cell (Fig. 4a) or T cell lineage (Fig. 4b,c) in blood collected at various post-surgical timepoints, we found virtually no cellular exchange had occurred three days after parabiosis surgery, similar to previous reports^46^,^47^. Only seven days following surgery, the percentage of GFP+ B cells and CD8+ or CD4+ T cells is similar between WT or GFP plasma, with complete chimerism achieved 14 days after surgery.

**Figure 5:**
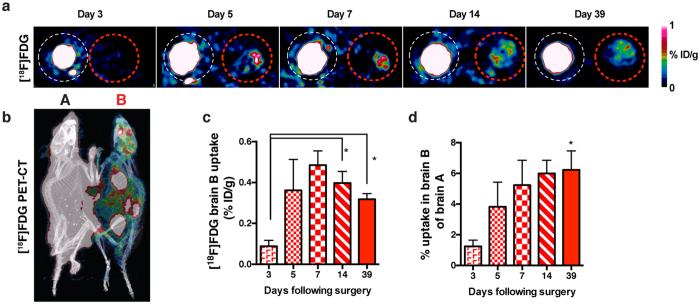
Timecourse of [^18^F]FDG transfer and brain uptake following parabiosis. (**A**) Representative coronal [^18^F]FDG-PET brain images from parabionts 1 h after tracer injection at indicated postsurgical timepoints outlined by white and red dashed circles around the injected “A” partner and non-injected “B” partner, respectively. **(B)** [^18^F]FDG-PET-CT reconstructed image of representative paired A and B mice. **(C)** Brain uptake of [^18^F]FDG, calculated as percentage of injected dose per gram body weight (% ID/g). 1-way ANOVA with Tukey’s post-hoc test (N = 4 pairs/timepoint). **(D)** Uptake in brain B as a percentage of brain A at various imaging timepoints. 1-way ANOVA with Dunnett’s test to compare timepoints vs. baseline (N = 4 pairs/timepoint; mean ± SEM). *P < 0.05.

Given that immune cells from one parabiont do not typically infiltrate the brain parenchyma of its partner^20^, we intravenously delivered a short-lived, radiolabeled glucose analog [^18^F]-FDG to overnight-fasted parabionts at different postsurgical timepoints to track uptake across the body and into the brain of the partner parabiont. As revealed by *in vivo* micro-positron emission tomography (micro-PET) one hour after injecting parabiont “A”, [^18^F]-FDG was detected in coronal brain sections of the non-injected (“B” partner) parabiont (Fig. 5d–f). [^18^F]-FDG uptake was significantly higher at later postsurgery imaging sessions, including days 14 and 39. Brain uptake of the tracer in parabiont B was approximately 6% of that seen in the injected mouse brain (Fig. 5g). A three-dimensional PET-CT video demonstrates more robust uptake of the metabolite in various organs in parabiont B (Fig. 5g), including liver, kidney, bladder, and heart (Supplementary Video).

## Discussion and Conclusion

Given the increased interest in parabiosis as a model for the study of complex physiology, including unexplored interactions between the CNS and peripheral immune compartments^6^, our goal was to characterize recovery and exchange parameters to inform the design and interpretation of experiments. By comparing the recovery of parabionts with that of mice given sham surgeries, we were able to identify recovery deficits as belonging to the surgery or the joining itself. We find that parabiosis decreases the velocity and activity of mice, though a stable baseline is ultimately achieved, likely as a result of the biomechanical constraints created by the procedure. It is also possible parabionts reach this lower baseline activity as a result of lower thermoregulatory requirements. Weight loss and subsequent stabilization to new weights were observed over a similar timecourse, consistent with previous work^21^,^46^. Various markers of general health, including those of liver and kidney function, were unchanged or returned quickly to baseline levels. Phosphorus levels, an indication of weight and nutritional fluctuation, seemed to reflect the weight change experienced following surgery in shams or parabionts. Furthermore, we observed surgery-associated hypercapnia following parabiosis, which may be ameliorated by more frequent saline supplementation or further nutritional and supportive care. Nesting, grooming, and motor coordination all recovered, albeit at delayed rates relative to mice given the sham surgery alone. Sleep behavior was unaffected by parabiosis at each timepoint following surgery, suggesting that the procedure is amenable to sensitive measures of brain activity, especially days to weeks after joining the mice. Power spectral density analysis of *in vivo* electrophysiological signals taken from the cortex of parabionts indeed revealed normal delta and theta rhythms during NREM and REM periods, respectively. Interestingly, following parabiosis, we observed alternating theta and delta-like rhythms during the waking state, a state normally dominated by voluntary, movement-associated theta rhythms. The broadened power pattern reflects motor adaptations made as the mice adjust to the physical constraints of parabiosis. Specifically, delta-like involuntary (passive waking) rhythms arose as a result of the partner mouse driving locomotion, whereas theta rhythms were observed as a result of movement initiated by the mouse undergoing EEG recording. Taken together, our behavioral and *in vivo* electrophysiological analysis of parabionts reveals that while the model limits overall locomotion, the procedure is not sufficient to alter important physiological behaviors, including nesting, grooming, motor coordination, and sleep.

The increasing exchange of lymphocytes followed a timecourse that was earlier than previously published^46^,^47^, perhaps due to differences in exchange afforded by the peritoneal model compared to epidermal parabiosis. Notably, the equilibration of chimerism was achieved at a time when many recovery parameters had also normalized. Given that previous cross-circulation studies have mostly relied on tracking labeled cells^13^,^46^, which do not appear to cross the blood-brain barrier^20^, we examined brain uptake and kinetics of a small molecule. [^18^F]-FDG-PET imaging revealed a surprisingly rapid exchange of a small molecule across the anastomosis and into the brain of the non-injected parabiont. The relatively low uptake for this metabolite may be a reflection of the tracer’s short half-life and rapid brain uptake in the injected mouse. While the exchange and uptake kinetics of other molecules may differ from that of [^18^F]-FDG, our results provide the first evidence that metabolites are indeed exchanged from one mouse to another and into the brain parenchyma. Systemic proteins of interest, such as those implicated in aging (CCL11, β2M, or GDF11) could be labeled using related approaches to examine brain entry from the blood.

For the design of future parabiosis experiments, we argue that the brain and other organs should be analyzed at a time not confounded by ongoing stress. Given that motor coordination and corticosterone levels normalize three and four weeks after parabiosis surgery, respectively, we recommend that mice be analyzed six weeks following surgery to allow for sufficient exchange while stress is minimal. Taken together, our results provide a framework with which to design and interpret parabiosis experiments for the study of complex interactions between the immune system and the brain.

## Additional Information

**How to cite this article**: Castellano, J. M. *et al*. *In vivo* assessment of behavioral recovery and circulatory exchange in the peritoneal parabiosis model. *Sci. Rep.* **6**, 29015; doi: 10.1038/srep29015 (2016).

## Supplementary Material

Supplementary Video

Supplementary Information

## Figures and Tables

**Table 1:** Serum chemistry/electrolytes and general health profiling of parabionts.

Metabolite	Baseline	4-day	15-day	28-day	28-day (sham)
Albumin	2.6 ± 0.12	2.7 ± 0.10	2.6 ± 0.10	2.5 ± 0.21	2.5 ± 0.08
Globulin	2.2 ± 0.12	2.3 ± 0.08	2.1 ± 0.05	2.7 ± 1.2	2.1 ± 0.15
A/G ratio	1.23 ± 0.10	1.20 ± 0.06	1.25 ± 0.06	1.20 ± 0.10	1.18 ± 0.15
Anion Gap	39.4 ± 5.57	36.9 ± 1.96	^**†**^42.2 ± 8.05	33.4 ± 1.27	30.3 ± 5.84
BUN	26 ± 4.5	24 ± 1.8	30 ± 11	21 ± 2.2	23 ± 0.84
Creatinine	0.60 ± 0	0.50 ± 0	0.53 ± 0.1	0.53 ± 0.06	0.54 ± 0.05
Total Protein	4.8 ± 0.16	^**##,††**^5.0 ± 0.15	4.8 ± 0.10	4.6 ± 0.21	4.7 ± 0.11
Ca^2+^	10.1 ± 0.33	10.5 ± 0.24	10.5 ± 0.57	7.9 ± 4.47	9.7 ± 0.44
Cl^−^	115 ± 4.27	115 ± 1.10	112 ± 1.71	118 ± 1.71	114 ± 1.52
CO_2_	12.7 ± 1.89	14.5 ± 1.39	14.4 ± 1.06	^*^16.0 ± 0.99	15.4 ± 1.91
Na^+^/K^+^ ratio	25.0 ± 3.73	22.6 ± 1.85	23.1 ± 2.18	26.3 ± 2.98	27.0 ± 2.35
Na^+^	162 ± 7.11	160 ± 1.10	162 ± 5.19	161 ± 3.16	156 ± 4.04
K^+^	6.4 ± 0.94	7.2 ± 0.55	7.1 ± 0.85	6.2 ± 0.68	5.8 ± 0.61
P	19.4 ± 2.67	^**^14.8 ± 1.54	^****^12.1 ± 1.64	^*^15.0 ± 0.93	^*^15.3 ± 2.09

Metabolite concentrations measured in serum collected terminally from presurgery baseline (N = 5–6), shams 28 days postsurgery (N = 4–5) or parabionts 4 (N = 5–6), 15 (N = 4), or 28 (N = 3–4) days postsurgery. Values are mean ± SD. Units are mg dL^−1^ for BUN, Creatinine, Ca^2+^, P; g dL^−1^ for total protein, albumin, and globulin; mmol L^−1^ for Na^+^, K^+^, Cl^−^, CO_2_. *, #, or † symbols indicate statistically significant difference compared to baseline, 4-week parabiont, or 4-week sham groups, respectively, assessed by 1-way ANOVA with Tukey’s post-hoc test across all groups. One, two, three, or four symbols indicate *P* < 0.05, *P* < 0.01, *P* < 0.001, or *P* < 0.0001, respectively.
