# Supplementary material for: In vivo assessment of behavioral recovery and circulatory exchange in the peritoneal parabiosis model
Source: Sci Rep. 2016 Jul 1;6:29015. doi: 10.1038/srep29015 (PMC4929497; doi:10.1038/srep29015)
Supplement: Supplementary Information [file srep29015-s2.pdf]

Supplementary information for: *In vivo assessment of behavioral recovery and circulatory factor exchange in the peritoneal parabiosis model*

Authors: Joseph M. Castellano, Mikael Palner, Shi-Bin Li, G. Mark Freeman, Jr., Andy Nguyen, Bin Shen, Trisha Stan, Kira I. Mosher, Frederick T. Chin, Luis de Lecea, Jian Luo, Tony Wyss-Coray

Supplementary Video 1: Shown in this video is an [^{18}F]FDG PET-CT imaging reconstructed video of parabionts 39 days after surgery, shown with respect to the scale of the non-injected parabiont. The video illustrates regional organ uptake of the tracer into the non-injected mouse as a result of parabiosis. Uptake signal intensity is shown on a scale from white/red (highest) to green/blue (lowest).
